# Humanizing harm: Using a restorative approach to heal and learn from adverse events

**DOI:** 10.1111/hex.13478

**Published:** 2022-03-23

**Authors:** Jo Wailling, Allison Kooijman, Joanne Hughes, Jane K. O'Hara

**Affiliations:** ^1^ School of Government, Te Ngāpara Centre for Restorative Practice Victoria University of Wellington Wellington New Zealand; ^2^ School of Nursing University of British Columbia Vancouver Canada; ^3^ Founder Harmed Patients Alliance UK; ^4^ School of Healthcare University of Leeds Leeds UK

**Keywords:** adverse events, incidents, investigations, patient safety, restorative

## Abstract

**Background:**

Healthcare is not without risk. Despite two decades of policy focus and improvement efforts, the global incidence of harm remains stubbornly persistent, with estimates suggesting that 10% of hospital patients are affected by adverse events.

**Methods:**

We explore how current investigative responses can compound the harm for all those affected—patients, families, health professionals and organizations—by neglecting to appreciate and respond to the human impacts. We suggest that the risk of compounded harm may be reduced when investigations respond to the need for healing alongside system learning, with the former having been consistently neglected.

**Discussion:**

We argue that incident responses must be conceived within a relational as well as a regulatory framework, and that this—a restorative approach—has the potential to radically shift the focus, conduct and outcomes of investigative processes.

**Conclusion:**

The identification of the preconditions and mechanisms that enable the success of restorative approaches in global health systems and legal contexts is required if their demonstrated potential is to be realized on a larger scale. The policy must be co‐created by all those who will be affected by reforms and be guided by restorative principles.

**Patient or Public Contribution:**

This viewpoint represents an international collaboration between a clinician academic, safety scientist and harmed patient and family members. The paper incorporates key findings and definitions from New Zealand's restorative response to surgical mesh harm, which was co‐designed with patient advocates, academics and clinicians.

## 1 BACKGROUND

Healthcare is not without risk. Despite two decades of policy and improvement efforts, the global incidence of harm remains stubbornly persistent.^1^ Investigating adverse events, particularly those leading to disability or death, provides the foundation of patient safety management systems globally.^1^ International policy approaches usually direct providers to undertake a transparent internal investigation of single events within a specified protocol. In some countries, such as the United Kingdom (UK) and Norway, the review of clusters of incidents is also supported. Regardless of the model applied, investigations usually seek to understand what happened and why, aiming to learn from events, improve systems and reduce the risk of reoccurrence.

The efficacy of the numerous investigation approaches used, and their impact on system learning is debated.^1^, ^2^ ‘Watershed’ public inquiries repetitively report ‘preventable’ deaths while illustrating devastating human impacts. Although these investigations provide a window into the scale of the problem, they are not without limitations. Often relying on retrospective analysis of written documents,^2^ the extent of the response of incident investigations and large‐scale inquiries, and their impact on system improvement, remains both challenging and under‐researched.^3^ Broader policy and reputational concerns, investigator preferences and embedded legal structures can impede the desired changes and the commitment to a ‘just and learning’ approach.^4^, ^5^

The role of those directly affected by the harm is usually limited to being a passive source of evidence, with the ‘testimony’ focused on the events themselves. Clearly, all those involved—patients, families and health professionals—provide credible information that is crucial to capture and learn from,^6^, ^7^ but this focus arguably prejudices the act of learning, over the experience of harm.^8^ Further, well‐intentioned investigative processes that pursue system improvement can create additional negative impacts.^7^, ^9^ In the aftermath of death or disability, and through the processes that follow—disclosure, investigation, resolution and change—not only are the human impacts of the initial event inadequately addressed but the experience of harm can be *compounded*.

In this paper, we propose that current investigative responses to adverse events can compound the harm for all the people involved—patients, families, health professionals and organizations—by neglecting to appreciate and respond to human impacts. We propose that the risk of compounded harm may be reduced when investigations provide the opportunity for healing alongside models that seek system learning, with the former having been consistently neglected. We argue that incident responses must be conceived within a relational as well as a regulatory framework, and that this—a restorative response—has the potential to radically shift the focus, conduct and outcomes of investigative processes.

## 2 WHAT IS COMPOUNDED HARM?

The assumption that system ‘learning’ and harm prevention are the only outcomes patients, families and health professionals desire from investigations is not in keeping with emergent evidence. In fact, there are increasing calls to acknowledge the wide‐ranging human impacts.^8^, ^9^, ^10^, ^11^ When an incident occurs, the people receiving and providing healthcare are hurt, and their relationships are affected. If this harm is to be adequately addressed—and safety enhanced—we contend that well‐being must be restored, and trust and relationships rebuilt. Compounded harm arises when these human considerations are not attended to, resulting in shame, contempt, betrayal, disempowerment, abandonment or unjustified blame, which can intensify over time.^11^, ^12^ Public inquiries often illustrate the negative impacts of embedded investigative responses, including the erosion of public trust in institutions and relationships, and the diminishment of individual or community wellbeing.^3^, ^9^

Compounded harm can also be derived from the failure of a responsible party, to give account to a harmed party, for harm that occurs whilst providing or receiving care.^12^ We define a responsible party as ‘any individual, group or entity that has had a significant role to play in the occurrence of the harm and/or the resulting reparative and preventative actions’.^12^ We submit that taking responsibility is not the same as accepting culpability; rather it is *a validating act* that can dignify all parties and may also be received as a demonstration of professional duty. The endurance of retributive approaches to investigations is a barrier to responsibility taking and is concerning given the evidence that health systems are complex and dynamic, and that events involve multiple people and systems.^13^

## 3 HOW DOES COMPOUNDED HARM ARISE?

Safety investigations are increasingly characterized by civil litigation and the criminalization of human error, despite assurances from safety scientists that individuals are rarely solely culpable.^7^ The actors involved in an incident are usually assigned roles more familiar in legal systems than safety critical industries. Typically, these roles are an initial ‘victim’, usually the patient or family member, and a ‘perpetrator’, a person, organization or regulator perceived to have caused the harm.

The adversarial conditions and entrenched positions of lengthy investigations usually prevent opportunities to bring patients, families and health providers together.^4^, ^14^ Ultimately, those closest to the incident lose their voice as assigned ‘advocates’ adopt the role of storyteller, and the narrative is shaped within frameworks concerned with system learning, litigation or reputation. Compounded harm can feel worse than the original injury, especially when people feel unheard or invalidated, and for some results in mental illness or suicidal ideation.^9^, ^11^, ^15^ These conditions prevent healing, defined as the restoration of wellbeing, relationships and trust.

Keeping people apart compounds harm because dialogue is necessary for healing. The wellbeing of injured patients and families suffers as the quality of therapeutic relationships is diminished and their experiences minimized.^9^, ^15^ Health professionals may experience distress as they lose their identity as healers, face ‘moral injury’ or are unable to express feelings of shame or remorse.^11^, ^16^ Public institutions can also lose the trust of the people they serve.^12^ Further, the often formal, distancing language associated with written reports and legal documents lacks the compassion of empathetic discourse.

Perhaps most importantly, relationships cannot be restored when trust in the fundamental, explicitly stated values and policy commitments are contradicted by lived experiences. A restorative response is required to repair substantive losses, employ a fair and transparent process of resolution and address the psychological needs of acknowledgement, respect and dignity of all the people involved.

## 4 WHAT IS A RESTORATIVE RESPONSE TO HARM?

Restorative responses belong to the collaborative, nonadversarial ‘Alternative Dispute Resolution’ (ADR) pathways that seek to function as an alternative to the formal system. Established pathways in international health settings incorporate approaches used in civil litigation, such as negotiation and mediation. While they share some common features with a restorative approach, each ADR pathway is distinguished by the practices, underpinning principles and values, and the outcomes sought.^12^, ^17^ The key differences are outlined in Table 1.

**Table 1:** The differences between current ADR approaches and a restorative response

Response	Underpinning values and principles	Procedure	Practices	Outcomes sought
Communication and resolution programme/open disclosure/Duty of candour (US, UK, Australia, Canada)	Transparency Learning Accountability Resolution	Procedure is predetermined. Hospitals and liability insurers disclose adverse events to patients; investigate; explain what happened; apologize; and in cases where substandard care caused harm, proactively offer compensation.	The people affected by the event are often represented by a proxy (lawyer, hospital manager). Mediation Arbitration Formal legal process (e.g., civil claims) Incident investigation	Reduce the number of malpractice claims and associated costs Legal agreement Formal apology System learning Compensation
Restorative response (NZ)	Active participation, respectful dialogue, truthfulness, responsibility‐taking, empowerment, equal concern	Procedure is codesigned by all the parties (patients, families, clinician and organization) and is underpinned by a restorative inquiry framework. Disclosure is expected from multiple actors.	Ideally, all the people affected by the event come together with the help of a skilled facilitator. Restorative conversations Facilitated meetings Circles Storytelling Actions captured in a shared document	To address harms, meet justice needs, restore trust and promote repair for all the people involved. An apology that meets people's needs (can include compensation) Healing and learning

Abbreviation: ADR, alternative dispute resolution.

Established ADR pathways are common in Australia, Canada, the United Kingdom and the United States. Approaches, such as ‘Communication and Resolution Programmes’ (CRPs) and ‘open disclosure’ focus on early transparent communication with harmed patients and families, complaint resolution and compensation when appropriate. While information exchange is understood to be crucial for learning, improvement and resolution, a paradigm based purely on information exchange provides no incentive or mechanism for building relationships or understanding one another.^18^, ^19^ Further, CRP programmes usually seek to reduce liability costs and the emphasis on financial risk may also limit their potential to respond to the human impacts.^20^ Research examining the patient and family experience of CRPs concluded that development should focus on nonadversarial communication, involvement of the treating clinician and ‘restorative competency’—defined as *‘*listening to patients stories without interrupting… to foster understanding and restore trust’.^21^

In contrast to approaches that promote disclosure, communication and resolution, restorative responses are fundamentally relational in nature. They appreciate that human relationships are at the core of the human experience of the world, are fundamental to human wellbeing and are implicated in our healing. We define a restorative response to an adverse event as:

*A voluntary, relational process where all those affected by an adverse event come together in a safe and supportive environment, with the help of skilled facilitators, to speak openly about what happened, to understand the human impacts, and to clarify responsibility for the actions required for healing and learning.*

**Figure 1:**
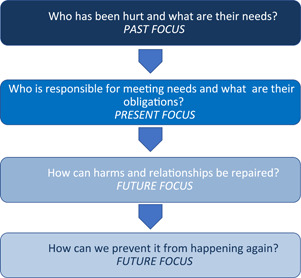
Restorative inquiry framework

The relational principles, values and goals strive to create open, trusting and respectful relationships that can help to prevent, mitigate or respond to harm. They include active participation, respectful dialogue, truthfulness, accountability, empowerment and equal concern for all the people involved.^9^, ^17^ The goal of a restorative response is to restore well‐being and relationships alongside understanding what happened. Accordingly, the dialogue is guided by a concern to address *harms*, meet *needs,* restore *trust* and promote *repair* for all involved.^9^, ^17^ Empathetic, respectful dialogue is achieved by bringing people together in a safe environment in face‐to‐face dialogue to answer the four questions of a restorative inquiry (Figure 1).

The questions asked in restorative inquiry support listening to understand and uncover the justice needs of the people involved. Justice needs are not synonymous with punishment. Rather, they encompass a holistic and caring approach that results in meaningful action for all the people affected. It is important to note that meeting justice needs is often a shared responsibility that requires access to diverse expertize and community support alongside the assistance offered by an investigator or team. Table 2 depicts some of the justice needs identified for harmed patients and families, health professionals and teams and the health provider and regulator during the restorative response to surgical mesh harm commissioned by New Zealand's Ministry of Health.^9^

**Table 2:** Examples of Justice needs identified during New Zealand's restorative response to surgical mesh harm^12^

Justice need	Patient/family	Health professional/team	Health provider/regulator
*Substantive* The actual harms that need to be remedied	Compensation Trauma counselling Peer support Childcare Meaningful apology Transport	Annual leave Trauma counselling Peer support	Reduce the likelihood of recurrence Make recommendations that will improve system safety Maintain public trust
*Procedural* The process of interacting, communicating, and making decisions about the harms	A just response where one can speak openly and honestly without fear of retribution Dialogue with parties identified as responsible e.g., clinicians, chief executive An advocate able to provide specialist advice and support Emotional support	A just response where one can speak openly and honestly without fear of retribution Dialogue with parties identified as responsible e.g., other clinicians, chief executive, professional bodies Open disclosure An advocate to provide specialist advice and support Emotional support	‘System learning’ within a ‘restorative just culture’ Meet regulatory requirements Open disclosure Dialogue with parties identified as responsible e.g., professional bodies, government agencies and policy makers
*Psychological*	To be heard and have their experience validated	To be heard and have their experience validated	To trust in the confidential nature of open conversations (e.g., not to be vilified in the press)
The way one is acknowledged, respected and treated throughout the process, ensuring those affected can honestly communicate their differences, concerns and potential similarities with each other in a safe way			
		Restoration of trust and confidence in therapeutic relationships	
	Restoration of trust and confidence in therapeutic relationships		
		To trust the confidential nature of open conversations	
	To trust the confidential nature of open conversations		

## 5 HOW MIGHT A RESTORATIVE APPROACH REDUCE COMPOUNDED HARM?

A restorative approach includes all the affected parties because they are best placed to explore what happened and make suggestions about how to promote restoration and mitigate future risks. This is a far more comprehensive and complex response than one which seeks to identify a victim, a perpetrator and a punishment; or indeed, one which simply assumes that system learning is the overwhelming priority. It has the potential to result in a meaningful apology because of the focus on essential apology characteristics; respectful dialogue, acknowledgement of responsibility and actions that address justice needs.^15^

A restorative approach uses specific practices that aim to create the conditions for psychological safety so that multiple perspectives of an incident can be understood through storytelling. Telling one's personal story of trauma has certainly been shown to have a range of cathartic effects,^22^ and there is tentative evidence that being able to choose how, to whom and how often to share a story of healthcare harm in a restorative process is a validating and dignifying experience for most people.^12^ A strength of the approach is a procedural adaptation, meaning emergent justice needs can be responded to as the story unfolds.^12^

The empathy elicited in dialogic exchanges between harmed patients, families and responsible parties is a powerful intrinsic motivator for learning, action and behaviour change.^12^ Restorative practices include affective statements, facilitated meetings between two parties or ‘Circle’ processes that may be used to establish group norms and respond to harm when there are larger groups involved. A Circle process involves a structured and intentional conversation in which people sit in a circle, and sequentially respond to questions posed by a facilitator.^9^ Both facilitated meetings and Circle practices typically follow the restorative inquiry framework.

To date, few studies have investigated restorative approaches within healthcare settings despite evidence for their utility across several domains.^23^ However, recent studies evaluating the approach provide tentative evidence for therapeutic, social and economic benefits. For example, the implementation of a ‘restorative just culture’ at one NHS Trust in England aimed to ‘fundamentally change the response to incidents, patient harm and complaints’.^24^ The approach responded to poor staff engagement and focused on improving the worker experience of disciplinary processes, incidents and complaints; an evaluation concluded that a range of positive economic, workforce outcomes was associated with this approach.^24^ However, given the study design, findings should be interpreted cautiously. Further, we view the lack of inclusion of the patient and family voice as problematic, if the goal is to fundamentally change the response to healthcare harm for all involved.

New Zealand's restorative response to harm from surgical mesh was facilitated by restorative justice experts and co‐designed with all the affected parties, including harmed patients, clinicians and policy makers. Examples of how restorative principles and values underpinned the New Zealand approach are provided in Table 3. The approach was evaluated within a health impact assessment framework, using mixed methods, to examine people's experiences of the process and the immediate impacts. The researchers determined that a restorative response can meet the justice needs of most patients, families and responsible parties, concluding it should be provided alongside existing regulatory structures, policies and procedures.^12^

**Table 3:** Examples of how restorative principles and values underpinned the New Zealand approach^9^, ^12^

Principle	Practice examples
Process is voluntary	Participants are prepared for a facilitated meeting
	Consent to proceed agreed by all parties (including the facilitator)
	Confidentiality parameters agreed
Process is relational and designed to meet the needs of those impacted	Substantive, procedural and psychological needs of all parties clarified during preparation, e.g., who needs to be involved? How would people like to tell their story and to whom?
	Access to emotional support before, during and immediately after a meeting
Respectful communication	Ground rules established during preparation and start of the meeting
	Facilitators minimized interruption and ensured conversational turn‐taking
	Facilitators upheld the ground rules and interjected to reframe, redirect or remind participants of their commitments when required
	If required, facilitators supported private conversations to clarify and repair any perceived hurtful comments
Safe environment	Confidentiality rules agreed at the outset, e.g., what will be shared and with whom
	Emotional support and breakout rooms provided
	Practical/comfort needs attended to
Skilled facilitation	Experienced practitioners guided the co‐design, preparation, restorative process and debriefing
Responsible parties are involved	Responsible parties heard directly about the harm experience to identify individual and shared responsibilities
Participants have an equal voice	Circle processes and facilitated meetings supported a democratic structure that is psychologically safe and supports shared decision‐making
	Responsible parties asked to listen and reflect key themes
Collaborative decision‐making	Potential actions collectively agreed to by consensus
Outcomes documented and shared	Actions committed to documented in a shared public document
	Collaborative governance approach for implementation agreed by all parties

## 6 HOW MIGHT THE RESTORATIVE APPROACH SHAPE AND IMPROVE THE RESPONSE TO ADVERSE EVENTS?

A restorative approach will be novel to many people working in healthcare policy and practice settings. This section briefly describes some areas where a restorative approach might shape and improve the response to a range of formal investigative processes, for example, adverse events, safety reviews of multiple incidents and national inquiries.

First, taking a restorative approach alters the process of disclosure in which apology plays a central role. Studies conclude that an informal explanation and assurances that an investigation will follow do not reduce formal complaints, can be associated with an increased risk of litigation and do not respond to individual needs.^15^, ^25^ A restorative approach may offer a way forward because of the explicit focus on understanding both what happened, and the unique justice needs, before responding within a meaningful apology characterized by reparative and preventative action.

Second, in eliciting, understanding and acting on the range of needs arising from an adverse event, a restorative response is likely to reduce the level of compounded harm experienced by all the people affected. The evaluation of New Zealand's inquiry reveals that the potential of a restorative approach is dependent on several critical success factors that should be considered (Table 3), all of these being usual in the successful application in other sectors.^12^

Third, in hearing from all the affected parties, when combined with traditional investigation approaches, the storytelling involved in a restorative response has the potential to improve individual, organizational and system learning.^12^, ^26^ Uncovering multiple perspectives of an event and developing recommendations within a psychologically safe, restorative consensus‐building approach, may improve the quality of recommendations and support their implementation, which is often challenging.^27^, ^28^, ^29^

Swiftly responding to the justice needs created by physical and/or psychosocial injuries can support the restoration of wellbeing, to the extent that repair is possible. Arguably, a no‐fault approach to financial compensation could assist in meeting justice needs. It could also reduce the risk of compounded harm resulting from lengthy legal processes associated with the retributive approach.^30^, ^31^ In New Zealand, where no‐fault legislation is embedded, efficacy and experience are influenced by several factors. Access to psychological support, and how the legislation is interpreted and interacts with other complaints and disciplinary processes, is particularly relevant.^9^, ^12^

To successfully achieve the restoration of wellbeing, relationships and trust, requires the embedding of restorative values and principles within interdependent policies, collaborative governance structures and organizational cultures.^12^, ^24^, ^32^ The development of theory about what works for whom and how, and research that investigates the impact of contextual conditions is essential to develop policy that enables successful implementation.^9^

Evidence regarding how minority groups and other vulnerable people experience patient safety interventions is limited.^29^ Authentic partnership and cultural diversity are essential considerations during policy development, implementation and evaluation of restorative responses. Arguably, some countries have a cultural disposition towards the restorative approach (e.g., New Zealand, Canada, North America and Australia), because an important root of restorative philosophy is Indigenous wisdom.^33^ However, systemic racism and inequities have recently been highlighted within these health systems,^34^, ^35^, ^36^ and such countries have an obligation to protect tribal authority over Indigenous knowledge and unique practices. Further, the success of restorative initiatives in European criminal justice settings indicates there is a broader appeal.^37^ This is perhaps because a key goal of all restorative approaches is to preserve the dignity of all the people involved. It has been suggested that, regardless of the cultural context, humans experiencing conflict or trauma share a fundamental need for dignity, where one is seen and heard as though one matters.^38^

Finally, including the perspectives of all the parties affected by adverse events in the design and evaluation of processes is essential to understand the numerous impacts and may serve as a protective factor when harm inevitably occurs.^12^ Further, embedding restorative theory and practice in health professional education may build capability and assist practitioners to heal those affected by an adverse event, including themselves, their colleagues and their communities, alongside safety science that emphasizes system learning.

## 7 CONCLUSION

We argue for a new approach to responding to adverse events, to reduce compounded harm and potentially provide a healing experience for all those involved, as well as enhance the scope and scale of learning. However, despite emergent evidence for restorative approaches in healthcare, many questions and evidence gaps remain. Identification of the preconditions and mechanisms that enable success in global health systems and legal contexts is required if their demonstrated potential is to be realized on a larger scale.

At their heart, restorative approaches are owned, developed and led by the people who are most affected by an incident. We must therefore transcend the dominant focus of enforcing a just and learning culture. The policy must be co‐created by all those who will be affected by reforms and be guided by restorative principles. Ultimately, embedding healing alongside learning is a worthy goal that will likely unite and be embraced by patients, families, health professionals and policy makers.

## CONFLICT OF INTEREST

The authors declare no conflict of interest.

## Data Availability

No data are shared for the purposes of this viewpoint article.
